# Impact of COVID-19 pandemic on antifungal consumption: a multicenter retrospective analysis

**DOI:** 10.1186/s13054-022-04270-z

**Published:** 2022-12-13

**Authors:** Anne-Lise Bienvenu, Audrey Bestion, Pierre Pradat, Jean-Christophe Richard, Laurent Argaud, Céline Guichon, Sandrine Roux, Vincent Piriou, Carole Paillet, Gilles Leboucher, the Antifungal study group, Florence Ader, Frédéric Aubrun, Charlotte Besson, Valentine Bréant, Charlotte Doudet, Sophie Ducastelle-Leprêtre, Damien Dupont, Marie-Delphine Guillemin, Véronique Leray, Charline Miossec, Sylvie Paulus, Anne-Marie Rabain, Pauline Rascle, Thomas Schulz, Michel Tod, Florent Valour, Florent Wallet, Martine Wallon

**Affiliations:** 1 Service Pharmacie, Groupement Hospitalier Nord, Hospices Civils de Lyon, 103 Grande rue de la Croix-Rousse, 69004 Lyon, France; 2 ICBMS CNRS 5246, Campus Lyon-Tech La Doua, Université de Lyon, Lyon, France; 3 Département d’Information Médicale, Hospices Civils de Lyon, Lyon, France; 4 Centre de Recherche Clinique, Groupement Hospitalier Nord, Hospices Civils de Lyon, Lyon, France; 5 Service de reanimation medicale, Groupement Hospitalier Nord, Hospices Civils de Lyon, Lyon, France; 6 Service de medecine intensive-reanimation, Hôpital Edouard Herriot, Hospices Civils de Lyon, Lyon, France; 7 Service de reanimation chirurgicale, Groupement Hospitalier Nord, Hospices Civils de Lyon, Lyon, France; 8 Service des Maladies Infectieuses et Tropicales, Groupement Hospitalier Nord, Hospices Civils de Lyon, Lyon, France; 9 Service d’anesthésie-reanimation, Groupement Hospitalier Sud, Hospices Civils de Lyon, Lyon, France; 10 Service Pharmacie, Groupement Hospitalier Centre, Hospices Civils de Lyon, Lyon, France

**Keywords:** Antifungals, Consumption, Defined daily dose, COVID-19, Invasive fungal disease

## Abstract

**Background:**

In the context of COVID-19 pandemic, antifungal overuse may have occurred in our hospitals as it has been previously reported for antibacterials.

**Methods:**

To investigate the impact of COVID-19 on antifungal consumption, a multicenter retrospective study including four medical sites and 14 intensive care units (ICU) was performed. Antifungal consumption and incidences of invasive fungal diseases before and during COVID-19 pandemic, for non-COVID-19 patients and COVID-19 patients, were described.

**Results:**

An increase in voriconazole consumption was observed in 2020 compared with 2019 for both the whole hospital and the ICU (+ 40.3% and + 63.7%, respectively), whereas the incidence of invasive aspergillosis significantly increased in slightly lower proportions in the ICU (+ 46%). Caspofungin consumption also increased in 2020 compared to 2019 for both the whole hospital and the ICU (+ 34.9% and + 17.0%, respectively) with an increased incidence of invasive candidiasis in the whole hospital and the ICU but in lower proportions (+ 20.0% and + 10.9%, respectively).

**Conclusions:**

We observed an increased consumption of antifungals including voriconazole and caspofungin in our hospital during the COVID-19 pandemic and explained in part by an increased incidence of invasive fungal diseases in COVID-19 patients. These results are of utmost importance as it raises concern about the urgent need for appropriate antifungal stewardship activities to control antifungal consumption.

## Introduction

Co-infections in patients suffering from COVID-19 are a clinical reality. In a meta-analysis, 15.9% of SARS-CoV-2 patients had a bacterial infection on presentation or during the hospital stay, 3.7% a fungal infection and 6.6% another respiratory viruses [1]. The most common fungal co-pathogens were *Aspergillus*, followed by *Candida* and more rarely *Mucor* [1]. COVID-19-associated pulmonary aspergillosis (CAPA) was recently recognized as a clinical entity [2] and a raising concern considering that the mortality rate of CAPA in the ICU is estimated to be around 50% [3]. In this context, empirical use of antifungals without waiting for final evidence of fungal microbiology was considered as a therapeutic strategy in COVID-19 critically ill patients [4]. As a consequence, antifungal overuse may occur, as previously reported for broad-spectrum antibacterials[5].

In this context, alarm bells have been rung by authors arguing that COVID-19 drug practices will invite aggravation of antimicrobial resistance [6]. To our knowledge, no study has described antifungal consumption and incidence of invasive fungal diseases during the COVID-19 pandemic compared to a period without a pandemic. Thus, we collected antifungal consumption and incidences of invasive fungal diseases, before and during the COVID-19 pandemic, for non-COVID-19 patients and COVID-19 patients.

## Methods

This retrospective analysis of antifungal consumption before (January 1, 2019, to December 31, 2019) and during the COVID-19 pandemic (January 1, 2020, to December 31, 2020) was performed in a university hospital including four medical sites and 14 intensive care units (ICU). This study was part of an antifungal stewardship program implemented in our university hospital since 2017 from which the major aim is to promote the optimal use of antifungals. In 2019, institutional treatment guidelines, including guidelines for treatment of invasive aspergillosis, invasive candidiasis and mucormycosis, were available, as well as all diagnosis procedures for those invasive fungal diseases. All institutional guidelines and procedures were validated by the anti-infective committee before being disseminated to clinicians through the antifungal group and the Committee for Medicinal Products and Medical Devices. During the study period, major diagnostic, therapeutic and infection control standards remained unchanged.

Antifungal consumption of main systemic antifungals, including voriconazole, fluconazole and caspofungin, was included. Consumption of other systemic antifungals, non-systemic antifungals and other antimicrobials was not included. It was expressed in defined daily dose (DDD) according to the Anatomical Therapeutical Chemical Classification methodology and the WHO DDD values [7]. DDD is the assumed average maintenance dose per day for a drug used for its main indication. DDD was expressed per 1000 patient-days. Antifungal consumption was extracted from the Pharmacy Department in 2019 and 2020, for the whole hospital and the ICU.

Incidences of invasive fungal diseases, including incidences of invasive aspergillosis, invasive candidiasis, mucormycosis and cryptococcosis, were extracted from the Medicalization Program of the Information Systemfor 2019 and 2020, for the whole hospital and the ICU, for non-COVID-19 and COVID-19 patients. European Organization for Research and Treatment of Cancer definitions of invasive fungal diseases were used by clinicians to support the diagnoses of invasive fungal diseases. This study was conducted in accordance with the Declaration of Helsinki and national and institutional standards. Ethical clearance was obtained from the Hospices Civils de Lyon scientific and ethics board as part of the Hospices Civils de Lyon Global COVID Research Initiative (2019–2022). Electronic records were under the auspice of the French National Committee for Data Protection and Freedom of Information.

Variables were presented with numbers and percentages. Incidence rates in 2019 and in 2020, in the whole hospital and in the ICU, for non-COVID-19 and COVID-19 patients, were compared using the Poisson distribution. *P* value < 0.05 was considered statistically significant. R-4.0.2 software (R Foundation for Statistical Computing, Vienna, Austria) was used for statistical analyses.

## Results

Consumption of systemic antifungals was increased by 22.2% in the whole hospital and 14.8% in the ICU (Table 1). The biggest increase in antifungal consumption from 2019 to 2020 was noted for voriconazole in both the whole hospital and the ICU (+ 40.3% and + 63.7%, respectively). Increased consumption of caspofungin was also observed in 2020 compared to 2019 in both the whole hospital and the ICU (+ 34.9% and + 17.0%, respectively). Increased consumption of fluconazole was noted only in the whole hospital (+ 16.2%) with consumption in the ICU remaining stable for the period (− 1.2%).

**Table 1:** Antifungal consumption including voriconazole, caspofungine and fluconazole, expressed in defined daily dose (DDD) per 1000 patient-days, in 2019 and 2020

Antifungal	2019 (DDD/1000 patient-days)	2020 (DDD/1000 patient-days)	Variation in DDD/1000 patient-days (%)
*Systemic antifungals*			
Whole hospital	40.1	49.0	+ 22.2
ICU	170.9	196.2	+ 14.8
*Voriconazole*			
Whole hospital	6.2	8.7	+ 40.3
ICU	29.8	48.8	+ 63.7
*Caspofungine*			
Whole hospital	4.3	5.8	+ 34.9
ICU	31.2	36.5	+ 17.0
*Fluconazole*			
Whole hospital	21	24.4	+ 16.2
ICU	84.3	83.3	− 1.2

*DDD* defined daily dose, *ICU* intensive care unit

In 2019 and 2020, incidences of invasive fungal diseases were higher in the ICU compared to the whole hospital, including invasive aspergillosis and invasive candidiasis (Table 2). Moreover, we demonstrated that overall incidence of invasive fungal diseases was comparable in the whole hospital in 2019 and 2020 (*p* = 0.265), whereas in the ICU, a 23.5% increase in incidence of invasive fungal diseases was observed in 2020 compared to 2019 (*p* = 0.003). If we analyzed the type of invasive fungal diseases, the incidence of invasive candidiasis significantly increased by 20.0% in the whole hospital in 2020 (*p* = 0.003), whereas the incidence of invasive aspergillosis remained stable for the period (*p* = 0.261). Of note, the incidence of invasive mucormycosis was decreased by 66% in 2020 in the whole hospital (*p* < 0.001). In the ICU, incidence of invasive aspergillosis was increased by 46% (*p* < 0.001), whereas the incidence of invasive candidiasis remained stable. Taking into account the COVID status of patients, we demonstrated a 78.0% increase in incidence of invasive fungal diseases with an incidence rate ratio of 4.64 in the whole hospital for COVID-19 patients compared to non-COVID-19 patients (*p* < 0.001), including invasive aspergillosis and invasive candidiasis. A 59.5% increased incidence of invasive fungal diseases with an incidence rate ratio of 2.52 was observed in the ICU for COVID-19 patients (*p* < 0.001), including invasive aspergillosis, but not invasive candidiasis.

**Table 2:** Incidences of fungal diseases in the whole hospital and in intensive care units, in 2019 and in 2020, for non-COVID-19 and COVID-19 patients

Year	2019			2020		
	Number of patients	Number of patients	Variation (%) 2019 vs 2020 (*p* =)	Number of COVID-19 patients	Number of non-COVID-19 patients	Difference (%) COVID-19 vs non-COVID-19 patients (*p* =)
	*n* = 402,426 *n* (%)	*n* = 360,514 *n* (%)		*n* = 5650 *n* (%)	*n* = 354,864 *n* (%)	
*Whole hospital*						
Invasive fungal diseases	892 (0.22)	843 (0.23)	+ 4.3 (*p* = 0.266)	58 (1.0)	785 (0.22)	+ 78.0 **(*p* < 0.001)**
Invasive aspergillosis	341 (0.09)	279 (0.07)	− 28.6 (*p* = 0.261)	38 (0.67)	241 (0.07)	+ 89.6 **(*p* < 0.001)**
Invasive candidiasis	502 (0.12)	540 (0.15)	+ 20.0 **(*p* = 0.003)**	20 (0.35)	520 (0.15)	+ 57.1 **(*p* < 0.001)**
Invasive mucormycosis	38 (0.009)	12 (0.003)	− 66.0 **(*p* = 0.002)**	0 (0)	12 (0.003)	NA (*p* = 1)
Cutaneous mucormycosis	4 (0.001)	2 (0.0006)	− 40.0 (*p* = 0.501)	0 (0)	2 (0.0006)	NA (*p* = 1)
Cryptococcosis	7 (0.002)	10 (0.003)	+ 50.0 (*p* = 0.344)	0 (0)	10 (0.003)	NA (*p* = 1)

	*n* = 22,255 *n* (%)	*n* = 20,344 *n* (%)		*n* = 1418 *n* (%)	*n* = 19,121 *n* (%)	
*Intensive care units*						
Invasive fungal diseases	290 (1.3)	336 (1.7)	+ 23.5 **(*p* = 0.003)**	53 (3.7)	283 (1.5)	+ 59.5 **(*p* < 0.001)**
Invasive aspergillosis	61 (0.27)	98 (0.5)	+ 46.0 **(*p* < 0.001)**	40 (2.8)	58 (0.3)	+ 89.3 **(*p* < 0.001)**
Invasive candidiasis	218 (0.98)	230 (1.1)	+ 10.9 (*p* = 0.129)	13 (1.1)	217 (1.1)	0 (*p* = 0.455)
Invasive mucormycosis	7 (0.03)	4 (0.02)	− 33.3 (*p* = 0.453)	0 (0)	4 (0.02)	NA (*p* = 1)
Cutaneous mucormycosis	4 (0.02)	1 (0.005)	+ 75.0 (*p* = 0.246)	0 (0)	1 (0.005)	NA (*p* = 1)
Cryptococcosis	0 (0)	3 (0.01)	NA (*p* = 1)	0 (0)	3 (0.02)	NA (*p* = 1)

Bold is used for* p*-values < 0.05

*NA* not applicable

## Discussion

We investigated antifungal consumption before and during the COVID-19 pandemic and incidences of invasive fungal diseases. During the first year of the COVID-19 pandemic, we observed increased consumptions of voriconazole and caspofungin in the whole hospital and in the ICU compared to 2019. Additionally, we showed increased incidences of invasive fungal diseases in the whole hospital and in the ICU in 2020, as a consequence of increased incidences of invasive fungal diseases in COVID-19 patients. COVID-19 was identified as a risk factor for invasive aspergillosis and invasive candidiasis in the whole hospital and a risk factor for invasive aspergillosis in the ICU. It should be noted that from July 2020, COVID-19 patients received, for acute respiratory distress syndrome in addition to standard of care, early dexamethasone therapy well known as a risk factor for invasive fungal diseases. Given that COVID-19 was associated with an increased risk for invasive aspergillosis in the ICU, this information was added in our institutional guidelines for invasive aspergillosis and clinicians were informed about this important feature when treating COVID-19 patients. Of note, a decrease in invasive mucormycosis incidence was observed in 2020 compared to 2019 that may be the consequence of a decrease in trauma cases during COVID shutdowns, as well as a reduction in haematopoietic stem cell transplantations in 2020.

As a consequence of increases in incidences of invasive fungal diseases in our hospital, consumptions of voriconazole and caspofungin increased in the whole hospital and in the ICU from 2019 to 2020, but sometimes in higher proportions than expected when compared with incidences of invasive aspergillosis or invasive candidiasis. The identification of COVID-19 as a risk factor for invasive fungal diseases, including invasive aspergillosis and invasive candidiasis, and the subsequent use of antifungals as empirical treatments for COVID-19 patients in our hospital [4], may explain the overuse of voriconazole and caspofungin during the first year of the COVID-19 pandemic. Of importance, the increased use of antifungals in 2020 may have decreased the occurrence of invasive fungal diseases, thus explaining the higher increases in percentages of antifungal consumption compared to incidences of invasive fungal diseases. An increase in antifungal consumption during the COVID-19 pandemic was previously reported [8], whereas a recent analysis of antimicrobials consumption worldwide during COVID-19 reported a slight nonsignificant decrease in consumption of antifungals [9]. Of note, these reports did not compare antifungal consumption with incidences of invasive fungal diseases.

This study had some limitations. We used data from Medicalization Program of the Information System that were possibly not exhaustive. We focused the analysis on caspofungin, fluconazole and voriconazole, the most used antifungals in our hospital during COVID-19 pandemic. In this retrospective study, it is not possible to assess the impact of empirical treatments on incidence of invasive fungal diseases.

Our results are of importance as they helped define appropriate antifungal stewardship activities to manage invasive fungal diseases in COVID-19 patients and control antifungal consumption with the aim of containing antifungal resistance in our hospital. Indeed, an increase in multidrug-resistant organisms during the COVID-19 pandemic was reported including pan-echinocandin-resistant *Candida glabrata* and multitriazole-resistant *Aspergillus fumigatus* [10]. The cause is said to be multifactorial, particularly a high rate of antimicrobial agent use in COVID-19 patients despite a relatively low rates of co-infections. Of course, the impact on resistance may vary according to the specific policies followed by different countries and hospitals. Lastly, antimicrobial policies and appropriate stewardship interventions including early reevaluation of empirical antifungal treatments are urgently needed to manage resistance in the COVID-19 era.

## Data Availability

All data generated or analyzed during this study are included in this published article.

## Abbreviations

CAPA: COVID 19-associated pulmonary aspergillosis
DDD: Defined daily dose
ICU: Intensive care unit

## References

[1] Alhumaid S, Al Mutair A, Al Alawi Z, Alshawi AM, Alomran SA, Almuhanna MS (2021). Coinfections with bacteria, fungi, and respiratory viruses in patients with SARS-CoV-2: a systematic review and meta-analysis. Pathogens.

[2] Koehler P, Bassetti M, Chakrabarti A, Chen SCA, Colombo AL, Hoenigl M (2021). Defining and managing COVID-19-associated pulmonary aspergillosis: the 2020 ECMM/ISHAM consensus criteria for research and clinical guidance. Lancet Infect Dis.

[3] Singh S, Verma N, Kanaujia R, Chakrabarti A, Rudramurthy SM (2021). Mortality in critically ill patients with coronavirus disease 2019-associated pulmonary aspergillosis: a systematic review and meta-analysis. Mycoses.

[4] Bienvenu A-L, Bleyzac N, Richard J-C, Leboucher G (2020). No time for pending confirmation of invasive fungal disease in critically ill COVID-19 patients-think empirical treatment. Crit Care.

[5] Rawson TM, Moore LSP, Zhu N, Ranganathan N, Skolimowska K, Gilchrist M (2020). Bacterial and fungal coinfection in individuals with coronavirus: a rapid review to support COVID-19 antimicrobial prescribing. Clin Infect Dis..

[6] Afshinnekoo E, Bhattacharya C, Burguete-García A, Castro-Nallar E, Deng Y, Desnues C (2021). COVID-19 drug practices risk antimicrobial resistance evolution. Lancet Microbe.

[7] WHOCC - Definition and general considerations [Internet]. https://www.whocc.no/ddd/definition_and_general_considera/. Cited 17 Aug 2022.

[8] Mulet Bayona JV, Tormo Palop N, Salvador García C, Fuster Escrivá B, Chanzá Aviñó M, Ortega García P (2021). Impact of the SARS-CoV-2 pandemic in candidaemia, invasive aspergillosis and antifungal consumption in a tertiary hospital. JoF.

[9] Khouja T, Mitsantisuk K, Tadrous M, Suda KJ (2022). Global consumption of antimicrobials: impact of the WHO Global Action Plan on Antimicrobial Resistance and 2019 coronavirus pandemic (COVID-19). J Antimicrob Chemother.

[10] Lai C-C, Chen S-Y, Ko W-C, Hsueh P-R (2021). Increased antimicrobial resistance during the COVID-19 pandemic. Int J Antimicrob Agents.

